# An analysis of discrepancies between United Kingdom cancer research funding and societal burden and a comparison to previous and United States values

**DOI:** 10.1186/s12961-015-0050-7

**Published:** 2015-11-02

**Authors:** Ashley J. R. Carter, Beverly Delarosa, Hannah Hur

**Affiliations:** Biological Sciences Department, California State University, Long Beach, 1250 Bellflower Boulevard, Long Beach, CA 90804 USA

**Keywords:** Cancer, Research funding, Years of life lost, United Kingdom, United States

## Abstract

**Background:**

Ideally, the allocation of research funding for each specific type of cancer should be proportional to its societal burden. This burden can be estimated with the metric ‘years of life lost’ (YLL), which combines overall mortality and age at death.

**Methods:**

Using United Kingdom data from 2010, we compared research funding from the National Cancer Research Institute to this YLL burden metric for 26 types of cancers in order to identify the discrepancies between cancer research funding allocation and societal burden. We also compared these values to United States data from 2010 and United Kingdom data published in 2005.

**Results:**

Our study revealed a number of discrepancies between cancer research funding and burden. Some cancers are funded at levels far higher than their relative burden suggests (testicular, leukaemia, Hodgkin’s lymphoma, breast, cervical, ovarian, prostate) while other cancers appear underfunded (gallbladder, lung, nasopharyngeal, intestine, stomach, pancreatic, thyroid, oesophageal, liver, kidney, bladder, and brain/central nervous system). United Kingdom funding patterns over the past decade have generally moved to increase funding to previously underfunded cancers with one notable exception showing a converse trend (breast cancer). The broad relationship between United Kingdom and United States funding patterns is similar with a few exceptions (e.g. leukaemia, Hodgkin’s lymphoma, prostate, testicular cancer).

**Conclusions:**

There are discrepancies between cancer research funding allocation and societal burden in the United Kingdom. These discrepancies are broadly similar in both the United Kingdom and the United States and, while they appear to be improving, this is not consistent across all types of cancer.

**Electronic supplementary material:**

The online version of this article (doi:10.1186/s12961-015-0050-7) contains supplementary material, which is available to authorized users.

## Background

Cancer is estimated to kill over 8.2 million people annually, including an estimated 617,229 people in the United States and 157,849 in the United Kingdom in 2012 [1]. In order to address this peril, governments fund grants and institutions specifically devoted to cancer treatment research. Since financial resources are limited, it is in the best interest of government agencies to ensure this money is spent effectively and efficiently by devoting the resources to cases in which the greatest benefit is likely to be realized. This realization prompted the United States National Institutes of Health (NIH) to recommend that research effort be compared to societal burden when making funding decisions [2]. Nevertheless, studies explicitly comparing cancer research funding to societal burden are limited in number [3–12] and often group all types of cancer together or include other diseases with different etiologies which obscure comparisons – we restrict our analyses to comparisons of different types of cancer to facilitate these.

Previous reports indicate that the cancer research funding levels for different cancer types are inappropriately aligned with societal burden metrics in both the United Kingdom [3] and the United States [10]. Many specific types of cancer appear either over- or underfunded according to various metrics examined. Since one of the goals of a well-designed national cancer research program is to efficiently allocate resources, mismatches between research funding and societal burden should be minimised. This requires two steps, (1) choosing an appropriate burden metric or metrics with which to rate each cancer and (2) shifting research funding toward a distribution that exhibits equal expenditure with respect to the burden metric or metrics identified.

The first burden metric typically considered is overall mortality. The total number of deaths from each cancer is the easiest epidemiological data to record and interpret, but this presents an incomplete picture of the overall burden. For example, one may argue that the death of a child and the death of person aged 95 years, caused by the same particular cancer, carry different burdens. Most people would agree that the death of the child is the more regrettable situation and represents a larger loss. Health care analyses therefore often adjust the societal burden of each death by taking into account the age at which a person is killed by the cancer.

The total ‘years of life lost’ (YLL) is a simple metric that takes into account the age at death when considering the degree of burden [3]. YLL is the number of years longer the patient is expected to have lived in the absence of that cause of mortality. YLL is based on the remaining life expectancy which changes as an individual ages due to sex and age group considerations. For example, the death of a 95 year old would result in approximately 3 years lost (since such a person lives 3 additional years on average) whereas the death of a 50 year old would result in approximately 32 years lost (since such a person lives, on average, 32 additional years). The YLL is the total number of years lost calculated for all individuals of all ages and is therefore a more complete measurement of the cost or burden arising from deaths than total mortality. YLL values have been used in previous studies of societal burden from disease (e.g. [3, 10]).

Three related statistics can be useful for considering the burdens caused by these premature deaths. The ‘average years of life lost’ (AYLL) gives a value that represents the average severity of each individual death using the YLL concept, but does not include information regarding the number of cases. AYLL is calculated by dividing the overall YLL value by the total mortality value (equivalent to calculating an average of the remaining life expectancy values weighted by the number of deaths in each age group). The ‘years of life lost per incidence’ [10] divides the total YLL by the number of incidences, which serves to reduce the value for cancers that may kill people when they are very young (and therefore have very high AYLL values), but for which we already have very high treatment efficacies (e.g. testicular cancer). The ‘disability adjusted life-years’ (DALY) metric sums the YLL and discounts the remaining years experienced by survivors by estimating the reduction in the quality of any remaining years of life due to disabilities caused by the disease. The DALY is widely used, but presents problems in comparative studies due to the subjective nature of assigning quantitative values to the quality of life discounts [13, 14]. While we believe these three metrics can also be used to provide insight into the burden imposed by cancer, we will focus mainly on the YLL in our analyses because this measures an unambiguous overall societal burden and is free of subjective decisions regarding disabilities and quality of life.

We analyzed burden metrics for 26 different types of cancer in the United Kingdom and compared the values to the funding levels provided by the National Cancer Research Institute (NCRI) with the goal of identifying mismatches between research funding and societal burden, hereafter referred to as discrepancies. Such discrepancies can then be considered on a case-by-case basis to better understand the state of cancer research efficiency. To place this information in a broader context we compared current discrepancies in the United Kingdom to those previously identified [3] to determine whether funding allocation has improved with respect to equitability and we compared the United Kingdom discrepancies to those recently reported for the United States [10].

## Methods

Mortality, YLL, and AYLL data was obtained in the following manner. We obtained mortality values for 2010 [15] and life expectancy values for 2011 for each 5-year age group (i.e. 0–4, 5–9, …, 75–79, 80–85, 85+ years) for each type of cancer for individuals living in the United Kingdom from WHO data [16], with the exception of separate life expectancy values for ages 0–1 and 1–4, in which case the latter was used. Each YLL value was calculated by multiplying the mortality values by the remaining life expectancy values for each age category and summing across all of them. Separate male and female mortality and life expectancy tables were used to calculate sex-specific YLL values and were combined for an overall YLL.

Funding values were obtained in the following manner. United Kingdom research funding values for each type of cancer were obtained from the Cancer Research Database published by the NCRI [17]. This is the major governmental source of cancer research funding in the United Kingdom, analogous to the NIH in the United States. Although this data does not represent all cancer research funding in the United Kingdom, it does provide data comparable to NIH funding analyses in the United States [10] and previous NCRI-based studies [3]. The NCRI divides research into ‘all-sites’ and ‘non-all-sites’ categories. We omitted the values for ‘all-sites’ and focused on the specific categories or types of cancer. All calculations only include the funding values for these specific cancer types.

The listed categories for cancer type are not identical in the WHO and NCRI sources described above. Based on the most recent International Statistical Classification of Diseases and Related Health Problems (ICD-10) from the WHO, some NCRI funding categories were combined in order to match the WHO mortality categories. The adjustments made were (1) Oral Cavity, Lip and Pharynx combined and assigned to “C00-14, not C11”, (2) Anal, Rectal and Colon combined and assigned to “C18-21”, and (3) Brain and Central Nervous System combined and assigned to “C70-72”. This resulted in 26 different types of cancer. We neglected all other values for cancer mortality and funding and performed all analyses on these values, omitting very rare cancers and funding not specifically targeted to one of the 26 categories. The 26 categories account for the vast majority of cancer cases and over 91.49% of the NCRI funding dedicated to specific types of cancer.

Due to its high burden and relatively low funding levels, lung cancer represents an outlier in many statistical considerations. Lung cancer causes over twice as many deaths as the next most lethal cancer category (anal/rectal/colon accounting for 12% of deaths) despite receiving less funding than four other cancer types (anal/rectal/colon, breast, prostate, and leukaemia) and roughly the same funding as ovarian cancer (which kills less than an eighth as many individuals). Specifically, lung cancer accounts for 26% of cancer deaths while receiving only 6% of cancer research funding; this tends to obscure other relationships between funding and societal burden if it is included in analyses. Furthermore, since the main cause of lung cancer is well known (i.e. smoking) and because most individuals are not diagnosed until later in progression, relative to other types of cancer, the types of lung cancer studies funded (according to the Common Scientific Outline research classification system [18]) focus more on prevention (e.g. excise tax on tobacco, adopting smoke-free laws and policies, etc.) than medical treatment [19]. Due to the extreme nature of mortality and YLL values for lung cancer and the different manner with which research for treatments for this cancer is prioritized, separate analyses with and without lung cancer values were performed. Both sets of results are available through a combination of the results presented in the main text which omit lung cancer and those which include lung cancer made available as Additional file 1.

## Results

Table 1 presents the mortality, YLL, funding, and AYLL data for the 26 cancer types for which data was available from the NCRI and WHO sources. The mortality and funding values are directly reported and the YLL and AYLL values were calculated as described above. The table also provides the abbreviations that are used in Table 2 and the figures.

**Table 1:** Data used in analyses. Presents the raw and calculated data used in our analyses. This table presents the ICD-10 codes, cancer type name, abbreviation used throughout this paper, mortality, YLL, funding and AYLL data from the NCRI and WHO sources for the 26 cancers examined in our study. The mortality and funding values are directly from the sources described; the YLL and AYLL values were calculated as described in the text.

**ICD - 10**	**Full Cancer Name**	**Abbreviation**	**Mortality**	**YLL**	**Funding (in £)**	**AYLL**
C00-14, not C11	Oral Cavity, Lip, & Pharyngeal	Oral	2200	41,862	3,838,687	19.03
C11	Nasopharyngeal	Naso	129	2,774	61,982	21.50
C15	Esophageal	Eso	7616	119,251	5,431,750	15.66
C16	Stomach	Stom	4967	71,851	2,487,957	14.47
C17	Intestine	Int	422	6,893	210,583	16.34
C18-21	Anal, Rectal, & Colon	An/Rec/Col	16046	236,029	22,668,843	14.71
C22	Liver	Liver	3805	62,002	2,839,325	16.29
C23-24	Gallbladder	Gall	725	10,372	94,656	14.31
C25	Pancreatic	Panc	7917	124,069	4,368,188	15.67
C32	Laryngeal	Larynx	761	12,618	1,469,229	16.58
C33-34	Lung*	Lung	34926	548,930	11,847,782	15.72
C43	Melanoma	Mela	2204	42,840	4,933,515	19.44
C50	Breast	Breast	11575	219,254	42,027,686	18.94
C53	Cervix Uteri (Cervical)	Cerv	940	24,862	4,287,905	26.45
C54	Corpus Uteri (Endometrial)	Uter	1452	23,874	2,600,732	16.44
C56	Ovarian	Ovary	4183	76,148	12,169,672	18.20
C61	Prostate	Pros	10729	114,805	16,629,771	10.70
C62	Testicular	Test	75	2,733	1,811,845	36.43
C64	Kidney	Kid	3728	60,669	3,433,812	16.27
C67	Bladder	Blad	4914	59,490	3,524,027	12.11
C70-72	Brain & Central Nervous System	Br/CNS	3838	87,015	5,669,837	22.67
C73	Thyroid	Thyr	346	5,347	228,627	15.45
C81	Hodgkin’s Disease	H Lym	324	7,941	2,182,898	24.51
C82-85, C96	Non-Hodgkin’s Lymphoma	NHL	4459	69,975	8,238,159	15.69
C88 + C99	(Multiple) Myeloma	Myel	2762	39,257	4,568,972	14.21
C91-95	Leukemia	Leuk	4492	71,337	32,545,100	15.88
		Total:	135535	2,142,198	£ 200,171,540

**Table 2:** Burden and funding proportions. Presents several proportional burdens and research funding values and their ratios. For each cancer type, this table presents the proportions of overall mortalities, YLL and funding (2^nd^, 3^rd^, and 5^th^ columns, respectively) dedicated to research. The ratio of proportional YLL to proportional mortality (4^th^ column) indicates which cancers tend to kill younger victims. The ratio of proportional funding to proportional mortality and YLL (6^th^ and 7^th^ columns, respectively) indicates which cancers appear to be overfunded (values larger than 1.0) or underfunded (values smaller than 1.0) including data for lung cancer. The rightmost column (8^th^ column) recalculates the funding to YLL ratio after omitting lung cancer.

**Cancer Site**	**%Mortality**	**%YLL**	**%YLL/%Mortality**	**%Funding**	**%Funding/%Mortality**	**%Funding/%YLL**
Test	.06	.13	2.31	.91	16.36	7.10
Leuk	3.31	3.33	1.00	16.26	4.91	4.88
H Lym	.24	.37	1.55	1.09	4.56	2.94
Breast	8.54	10.24	1.20	21.00	2.46	2.05
Cerv	.69	1.16	1.67	2.14	3.09	1.85
Ovary	3.09	3.55	1.15	6.08	1.97	1.71
Pros	7.92	5.36	.68	8.31	1.05	1.55
NHL	3.29	3.27	.99	4.12	1.25	1.26
Larynx	.56	.59	1.05	.73	1.31	1.25
Myel	2.04	1.83	.90	2.28	1.12	1.25
Mela	1.63	2.00	1.23	2.46	1.52	1.23
Uter	1.07	1.11	1.04	1.30	1.21	1.17
An/Rec/Col	11.84	11.02	.93	11.32	.96	1.03
Oral	1.62	1.95	1.20	1.92	1.18	.98
Br/CNS	2.83	4.06	1.43	2.83	1.00	.70
Blad	3.63	2.78	.77	1.76	.49	.63
Kid	2.75	2.83	1.03	1.72	.62	.61
Eso	5.62	5.57	.99	2.71	.48	.49
Liver	2.81	2.89	1.03	1.42	.51	.49
Thyr	.26	.25	.98	.11	.45	.46
Panc	5.84	5.79	.99	2.18	.37	.38
Stom	3.66	3.35	.92	1.24	.34	.37
Int	.31	.32	1.03	.11	.34	.33
Naso	.10	.13	1.36	.03	.33	.24
Lung	25.77	25.62	.99	5.92	.23	.23
Gall	.53	.48	.91	.05	.09	.10

Table 2 presents the proportional values for the mortality, YLL and NCRI research funding for each cancer type and some calculated ratios. The ratio of YLL to mortality indicates those cancers that tend to kill younger victims. The last two columns present the ratio of NCRI research funding to the YLL values with and without the inclusion of lung cancer values and the table is ordered by these. Larger ratios in the rightmost three columns indicate cancer types overfunded relative to the overall funding levels and values below unity reflect cancers that are underfunded relative to their societal burden. The top six and bottom eight cancer types are the same when ordered by funding relative to either mortality or YLL while other cancers shift within the middle region. These ratios indicate research effort to societal burden discrepancies, but they do not indicate overall societal burden; for example, testicular cancer is by far the most overfunded type of cancer according to both criteria, but this arises from a very low number of testicular cancer mortalities.

The effect of including or omitting lung cancer is clear from comparing the last two columns of Table 2. As described in the Methods section, we believe that omitting lung cancer shows a clearer picture of the overall funding and prioritization patterns. The figures are therefore based on values omitting lung cancer data (i.e. using values from the last column in Table 2), but these same figures with lung cancer values included are available as Additional file 2 and reference to them will occasionally be made in the discussion.

**Figure 1:**
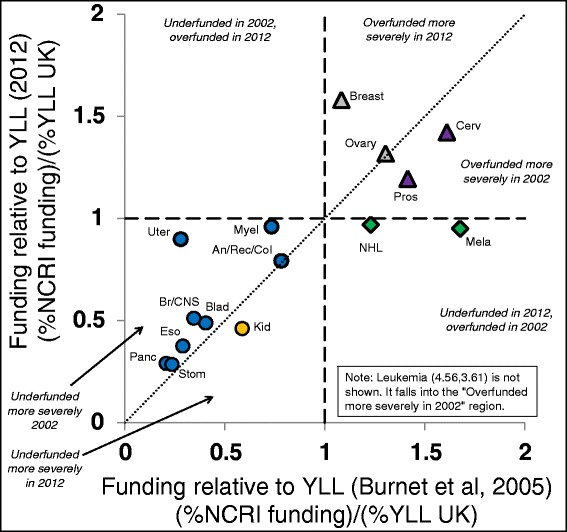
Funding versus years of life lost (YLL) over time in the United Kingdom. Presents the research funding to YLL ratios for the United Kingdom data in Table 2 on the Y-axis compared to 2002 data [3] on the X-axis. The dashed vertical and horizontal lines indicate points at which proportional funding is equal to proportional YLL. The dotted diagonal line indicates identical discrepancies during both time periods. Italicized labels are used to describe six zones within the figure that represent different combinations of patterns of discrepancy. Combinations of symbol shape and colour/shading indicate cancer types with similar types of funding discrepancies. Cancer types which are above the diagonal line to the left of the vertical dashed line indicate those that were previously underfunded receiving increased funding and cancer types below the diagonal line to the right of the vertical dashed line indicate those that were previously overfunded receiving reduced funding. Not all 26 cancer types are represented due to less comprehensive data for the older United Kingdom data set.

**Figure 2:**
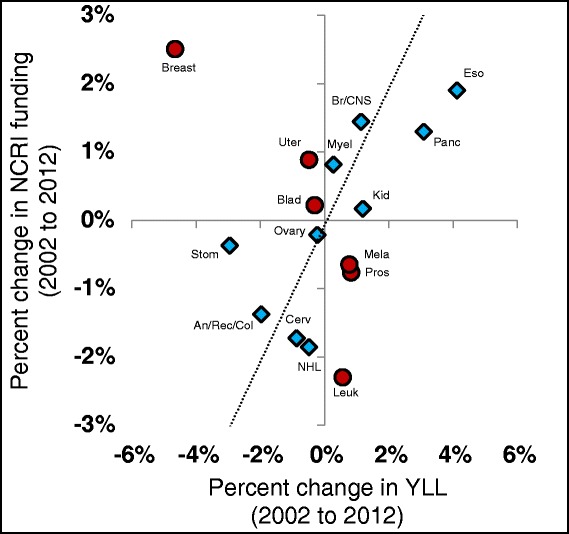
Funding and years of life lost (YLL) changes over time in the United Kingdom. Presents a comparison of the percentage differences in research funding (Y-axis) and YLL (X-axis) for the United Kingdom between this study and the values reported for 2002 [3]. Data points to the right of the vertical axis indicate cancers increasing in relative societal burden (and vice versa) while data points above the horizontal axis represent cancers receiving increased funding (and vice versa). Combinations of symbol shape and colour/shading indicate cancer types with concordant or discordant changes in funding and YLL. These values are relative YLL and funding so any increases in the relative YLL for a specific cancer type would not mean increases in absolute YLL, rather that the relative severity is increasing, most likely due to better improvements for other types of cancer. Not all 26 cancer types are represented due to less comprehensive data for the older United Kingdom data set.

Figures 1 and 2 compare the values obtained in this study to those reported in 2005 for the United Kingdom which were based on demographic data from 1994 and funding data from 2002 [3]; we will hereafter consider them to represent values indicative of the year 2002, which allows comparisons approximately corresponding to the changes over the past decade.

Figure 1 compares the research funding to YLL ratios for the current and 2002 United Kingdom data [3]. There is a significant relationship between these values (R^2^ = 0.904 with *P* = 1.6 × 10^−7^, ANOVA F test) suggesting that funding discrepancies are persistent rather than arising due to random fluctuations in funding cycles. There are still many overfunded and underfunded cancers and leukaemia presents an extreme case in which a very high degree of overfunding is apparent in both time periods (R^2^ = 0.638 with *P* = 3.5 × 10^−4^, ANOVA F test when omitting leukaemia). The figure indicates that these discrepancies are generally being reduced, as indicated by cancer types which are above the diagonal line for previously underfunded cancers and below the diagonal line for previously overfunded cancers. A majority of the cancer types that were underfunded in 2002 received increased funding (8 of 9) and a majority of cancer types that were overfunded in 2002 received reduced funding (5 of 7). Lung cancer was previously underfunded and continues to be so, but to a slightly lesser degree (Additional file 3: Figure S1).

Figure 2 compares the changes in research funding and changes in YLL for the United Kingdom since 2002 [3], expressed in relative terms. An increase in the relative YLL for a specific cancer type does not necessarily mean an increase in absolute YLL; instead, the relative severity is increasing, most likely due to better improvements for other types of cancer. Overall, there is an extremely weak and non-significant relationship between types of cancer that have increases in relative YLL and those with increases in funding (R^2^ = 0.016 with *P* = 0.645, ANOVA F test). However, this lack of a relationship is driven by breast cancer and an analysis omitting breast cancer results in a stronger and statistically significant relationship (R^2^ = 0.321 with *P* = 0.028, ANOVA F test). Overall, this figure indicates that, with the exception of breast cancer, funding is shifting toward cancers that lag behind in terms of effective treatments.

**Figure 3:**
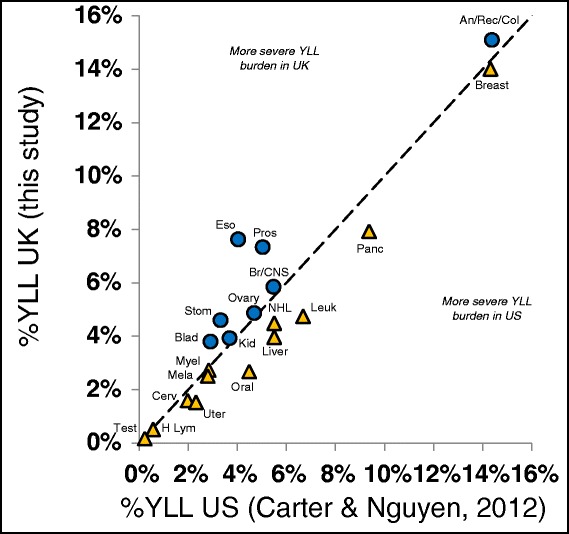
Years of life lost (YLL) values in the United Kingdom versus the United States. Presents the YLL burden values for the United Kingdom on the Y-axis compared to a recent report of the same cancer types for the United States [10]. Both data analysis are based on 2010 values. The dotted diagonal line indicates equal proportional YLL values in the two countries and residuals from this line indicate types of cancer for which the societal burdens differ. Not all 26 cancer types are represented due to less comprehensive data for the United States data set.

Figure 3 compares YLL values for the United Kingdom and United States [10]. There is a clear and significant relationship between the YLL values in the two countries (R^2^ = 0.882 with *P* = 8.7 × 10^−10^, ANOVA F test) as expected for two countries with so many cultural similarities. Oesophageal cancer presents the largest deviation from the pattern with a 1.89:1 ratio of relative YLL values.

**Figure 4:**
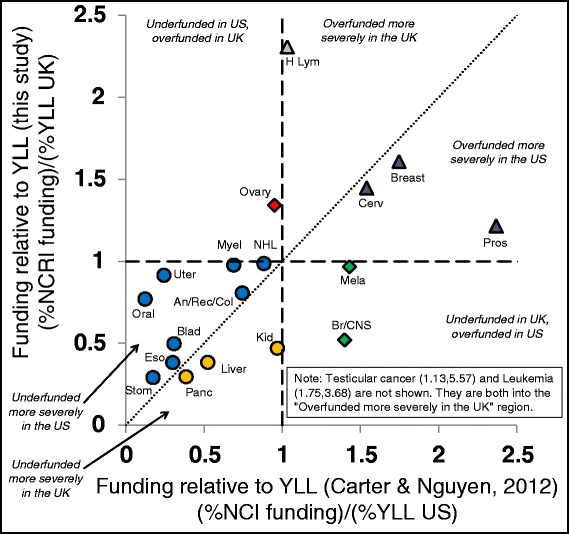
Funding versus years of life lost (YLL) in the United Kingdom versus the United States. Presents the research funding to YLL ratios for the United Kingdom data in Table 3 on the Y-axis compared to a recent report of the same cancer types for the United States [10] on the X-axis. Both data analyses are based on 2010 values. The dashed vertical and horizontal lines indicate points at which proportional funding is equal to proportional YLL. The dotted diagonal line indicates identical discrepancies in both countries. Italicized labels are used to describe six zones within the figure that represent different combinations of funding discrepancies. There is a generally similar pattern of ratios although testicular cancer and leukaemia represent outliers in which they are overfunded in both countries, but to a much higher degree in the United Kingdom than in the United States.

Figure 4 compares the research funding to YLL ratios for the United Kingdom and United States data [10]. There is a weak and almost significant relationship between these values (R^2^ = 0.165 with *P* = 0.0754, ANOVA F test) suggesting similar research discrepancies in the two countries. Testicular cancer and leukaemia represent outliers in which they are overfunded in both countries, although to a much higher degree in the United Kingdom; omitting these from the analysis results in a significant relationship between the discrepancy values (R^2^ = 0.285 with *P* = 0.0225, ANOVA F test). The pattern is quite scattered and a number of cancers exhibit very different discrepancies (i.e. brain/central nervous system, kidney, leukaemia, oral, uterine, prostate, Hodgkin’s lymphoma, testicular) although some of this arises due to statistical variation in cancer types for which absolute burden or funding is low (e.g. Hodgkin’s lymphoma, testicular). Five cancer types (bladder, oesophageal, liver, pancreatic, stomach) appear considerably underfunded in both countries as is lung cancer (Additional file 4: Figure S4).

## Discussion

Our results indicate several discrepancies between the relative levels of funding from the NCRI and societal burden for the United Kingdom. Table 2 shows that some cancers are funded at levels higher than their relative burden suggests (testicular, leukaemia, Hodgkin’s lymphoma, breast, cervical, ovarian, prostate), while other cancers appear underfunded (gallbladder, nasopharyngeal, lung, intestine, stomach, pancreatic, thyroid, oesophageal, liver, kidney, bladder, and brain/central nervous system).

Comparisons with older United Kingdom data [3] and recent United States data [10] also reveal interesting patterns. Changes in funding in the United Kingdom over the past decade have reduced, but not eliminated, these discrepancies (Figure 1). Funding also appears to have been appropriately moving toward cancer types that lag behind others in terms of recent improvements that have led to lower mortality rates (Figure 2) with the exception of funding for breast cancer, which is a notable counter example. The relative severities of each type of cancer are quite similar in the United Kingdom and United States (Figure 3) and the pattern of funding levels relative to YLL burden is broadly similar in both countries (Figure 4), although there are cancers that appear differently prioritized in terms of this ratio (e.g. brain/central nervous system, kidney, leukaemia, oral, uterine, prostate, Hodgkin’s lymphoma, testicular). In both the United Kingdom and the United States, bladder, oesophageal, liver, lung, pancreatic, and stomach cancers are considerably underfunded relative to their societal burden as measured by both mortality and YLL.

One possible cause of differences in cancer mortalities and funding discrepancies between the United Kingdom and United States arises from the fact that the United Kingdom practices comprehensive nationalized medicine which can have several effects. First, the United Kingdom has a larger focus on regular check-ups and preventative measures that may lead to higher chances of diagnosing cancers at earlier, more treatable, stages. This would decrease the YLL values preferentially for cancer types that respond to early treatment. Second, without a nationalized healthcare system, the United States likely collects less complete medical data for its citizens and we therefore expect more statistical noise in the United States YLL values, especially for rare cancers.

Consideration of AYLL values (Table 1) has been used as an alternative method to measure societal burden [3], but we find this method flawed. Although AYLL places more weight on younger people’s deaths, it ignores the total number of cases. For example, testicular cancer has an extremely high AYLL of 36.43 years, but less than 1% of the total adjusted funding, which may make it appear to be greatly underfunded, but testicular cancer has the lowest mortality rate within our data set of 26 cancer types with a total of only 75 deaths in 2010 and is, in fact, overfunded when using overall YLL. Another example is cervical cancer, which appears overfunded (2.14% of funding, 1.16% of YLL) which may have arisen from its unusually high AYLL value (26.45, second highest value in our data set) despite the presence of an effective preventive mechanism (i.e. the Human Papillomavirus vaccine and yearly pap smear screenings) [20]. For reasons like these, our recommendations focus on YLL instead of either total mortality or AYLL because of the potentially misleading nature of those metrics.

However, there are caveats to remain aware of when matching research funding and effort directly to YLL. As stated in the Materials and Methods section, it is arguable that for some cancer types (e.g. lung and cervical), available funding could be better spent on prevention rather than treatment. This is because the risk of developing these cancers arises primarily from behavioural actions (e.g. smoking and sexual intercourse for lung and cervical cancers, respectively) [21–23] and the right prevention campaign could decrease burden dramatically without the need to develop more effective treatments. Because our study focused primarily on research funding provided by the government and did not include the research efforts held by private and commercial entities, our analysis is a partial view of the overall research funding context. We expect that funding discrepancies may be much higher in private funding than in the governmental data; rare cancers may be completely neglected by commercial entities due to the very small potential for profit that may be realized by developing a treatment with such a small market. The use of societal burden as the sole metric also lacks consideration of the complexity of performing research. Factors, such as private sector efforts, positive feedback cycles and negative synergistic effects, can result in different conclusions regarding the desired relationship between a simple burden metric like YLL and research effort [10]. These other factors are very difficult to quantify, however, and concrete information on their effects and importance is currently lacking. Despite these issues, organizations such as the NCRI and NIH recommend using a funding approach based on health metrics such as the YLL [2, 3, 10].

Our recommendations are based on the ideal of a linear relationship between societal burden (mortality or YLL) and effort (research funding). The fit between these burdens and effort was also explored using a variety of other types of relationships (e.g. exponential, logarithmic, polynomial, etc.); only an exponential curve yielded a better fit and the improvements were extremely minor. The linear best-fit lines show that current funding more closely matches a relationship between funding and YLL than funding and mortality (R^2^ = 0.55 and R^2^ = 0.47, respectively, if we exclude lung cancer and R^2^ = 0.11 and R^2^ = 0.07, respectively, if lung cancer is included) although the fit with YLL is only marginally better than the fit with raw mortality.

Certain types of cancers exhibit unusual funding and YLL relationships and therefore warrant additional discussion. We observed that breast cancer appears to be overfunded, which we feel may be due to a high level of public awareness for this specific cancer type. Some other cancers suffer from very low public awareness which may account for underfunding; for example, nasopharyngeal, intestine, gallbladder, and thyroid cancers, which together account for 1.2% of all YLL, only receive 0.3% of the total research funding.

Some cancers may also be underfunded due to a social stigma, in which it is not socially normal or appropriate to talk openly about them and raise awareness or consider burden. The very private and personal parts of the body associated with bladder and anal/rectal/colon cancers may account for the relatively low level of funding for these cancers and why there do not appear to be as many charitable organizations addressing these types of cancers as there are for breast cancer for example.

Figure 1 shows several cases in which funding levels have gone from overfunded or underfunded to much more equitably funded (melanoma and non-Hodgkin lymphoma for the former and myeloma and uterine cancer for the latter). These changes may have been due to decision makers following previous recommendations to move resources from overfunded to underfunded cancers (Figure 2).

Counter-examples to this process appear in cancer types with strong psychological and identity components. The main exception to the improvement in allocation to burden ratios appears to be breast cancer, for which large decreases in proportional YLL over the past decade are paired with increases in proportional research funding. The disproportionately high levels of funding for breast cancer may arise from the same emotional and awareness factors that have led to the numerous charitable organizations devoted to this cancer. This more highly perceived burden may arise from the strong psychological attachment women feel for their breasts as an embodiment of their feminine gender which adds a societal burden not captured by mortality or YLL. Additionally, since breast cancer has more widely used and visible tests than other cancers for early tumour detection (e.g. self-exams, mammograms, etc.), awareness is raised each time a woman performs these tests. Similar psychological processes, when played out in men, may account for the relatively high rates of funding for prostate and testicular cancers.

Psychological and emotional factors can also be responsible for the underfunding of some cancers. It appears that lung cancer receives low level of research funding due to a blame-the-victim-attitude, in which the personal choice to engage in smoking is seen as the direct and exclusive cause of lung cancer [24]. Oral and liver cancers, being linked to tobacco and alcohol use, are also prone to this bias. The public may view the best solution to the burden imposed by these cancers as arising not from improved medical research, but from social and behavioural changes. This attitude seems inconsistent with funding for cervical cancer, however, which is overfunded based on relative YLL yet often arises due to behavioural choices (e.g. sexual activity). Melanoma is also similar in that it is highly preventable by managing unprotected sun exposure. Therefore, if policymakers are inclined to devote less research funding to cancers that arise partially as a product of poor voluntary behaviour, then cervical cancer and melanoma would be expected to also be underfunded. Alternatively, if policymakers decide that it is not ethically tolerable to devote reduced funding to cancers wherein victims may arise due to their risk-inducing lifestyle choices, then lung, liver and oral cancers should be given increased funding.

Figure 3 shows that the United Kingdom, in comparison to the United States, has a much higher proportional YLL value for oesophageal cancer (almost twice the relative YLL) with less dramatic elevations apparent for stomach, bladder and prostate cancers while exhibiting slightly lower relative burdens arising from leukaemia, liver, oral, and uterine cancers. The United Kingdom drinks almost four times as much black tea as the United States on a per kg per person ratio [25] and recent studies found that the excessive consumption of hot tea increased the burden arising from several gastrointestinal cancers (e.g. oesophageal, prostate, and bladder cancer) [26–28]. Although this pattern is far from conclusive, the presence of elevated relative YLL for these cancers of the digestive system in the United Kingdom relative to the United States does suggest that some aspects of diet and culture may be responsible.

Figure 4 shows that although both the United Kingdom and United States appear to greatly overfund research on testicular cancer and leukaemia, these discrepancies are more extreme in the United Kingdom. We do not offer an interpretation of this difference between the countries, but we note that the culture of science itself may foster discrepancies in funding in cases such as these. New research scientists are trained in research labs that have received training and will naturally continue their careers in the same sub-disciplines. The competitive nature of gaining research funding (which often requires evidence of previous success) can create feedback loops in which areas of research with demonstrated success result in more individuals trained in that field than in other fields. The pool of researching scientists then resembles the types of research that has been successful rather than those that may provide the biggest societal benefit. Perhaps successful discovery of treatments for testicular cancer (it has a better than 95% cure rate) and leukaemia have perpetuated research in these areas to such a degree that it now outpaces that justified from the current societal burden. To reduce these discrepancies arising from research focus inertia, we recommend that researchers and funding agencies be mindful of the potential benefits arising from supporting research in novel areas.

In general, we found broadly similar patterns of discrepancy between societal burden (as measured by YLL) and governmental research funding in both the United States and the United Kingdom and evidence that these discrepancies generally improved in the United Kingdom from 2000 to 2010. The comparison with the United States benefited from having a recent United States data analysis [10] with easily comparable values, but the comparison with the previous United Kingdom data [3] was less than ideal due to the lack of a consistent base year for their research funding and mortality values.

## Conclusions

Discrepancies between the amount of NCRI research funding and United Kingdom societal burden exist for various types of cancers; some cancers are funded at levels far higher than their relative YLL burden suggests (testicular, leukaemia, Hodgkin’s lymphoma, breast, cervical, ovarian, prostate), while others appear underfunded (gallbladder, nasopharyngeal, lung, intestine, stomach, pancreatic, thyroid, oesophageal, liver, kidney, bladder, and brain/central nervous system). These discrepancies are similar to both those that were present a decade ago in the United Kingdom [3] and those current in the United States [10], indicating that these discrepancies are persistent and widespread. Since increased funding has been shown to lead to increased results [29–31], shifting resources from those cancers that are overfunded to those that are underfunded may increase the overall efficiency of cancer research with respect to reducing societal burden.

## Additional files

Additional file 1:
**Analysis of the research funding to years of life lost ratios for United Kingdom data presented in Table** 2 **on the Y-axis compared to 2002 data [** 3 **] on the X-axis, including lung cancer values.** (PDF 89 kb) Additional file 2:
**Analysis of a comparison of the percentage differences in research funding (Y-axis) and years of life lost (X-axis) for the United Kingdom between this study and the values reported for 2002 [** 3 **], including lung cancer values.** (PDF 100 kb) Additional file 3:
**Analysis of the years of life lost burden values for the United Kingdom on the Y-axis compared to a recent report of the same cancer types for the United States [** 10 **], including lung cancer values.** (PDF 103 kb) Additional file 4:
**The research funding to years of life lost ratios for United Kingdom data presented in Table** 2 **on the Y-axis compared to a recent report of the same cancer types for the United States [** 10 **] on the X-axis, including lung cancer values.** (PDF 103 kb)

## Abbreviations

AYLL: Average years of life lost
DALY: Disability adjusted life-years
ICD-10: International Statistical Classification of Diseases and Related Health Problems
NCRI: National Cancer Research Institute
NIH: National Institutes of Health
YLL: Years of life lost
