# Supplementary figures and images for: An analysis of discrepancies between United Kingdom cancer research funding and societal burden and a comparison to previous and United States values
Source: Health Res Policy Syst. 2015 Nov 2;13:62. doi: 10.1186/s12961-015-0050-7 (PMC4629288; doi:10.1186/s12961-015-0050-7)

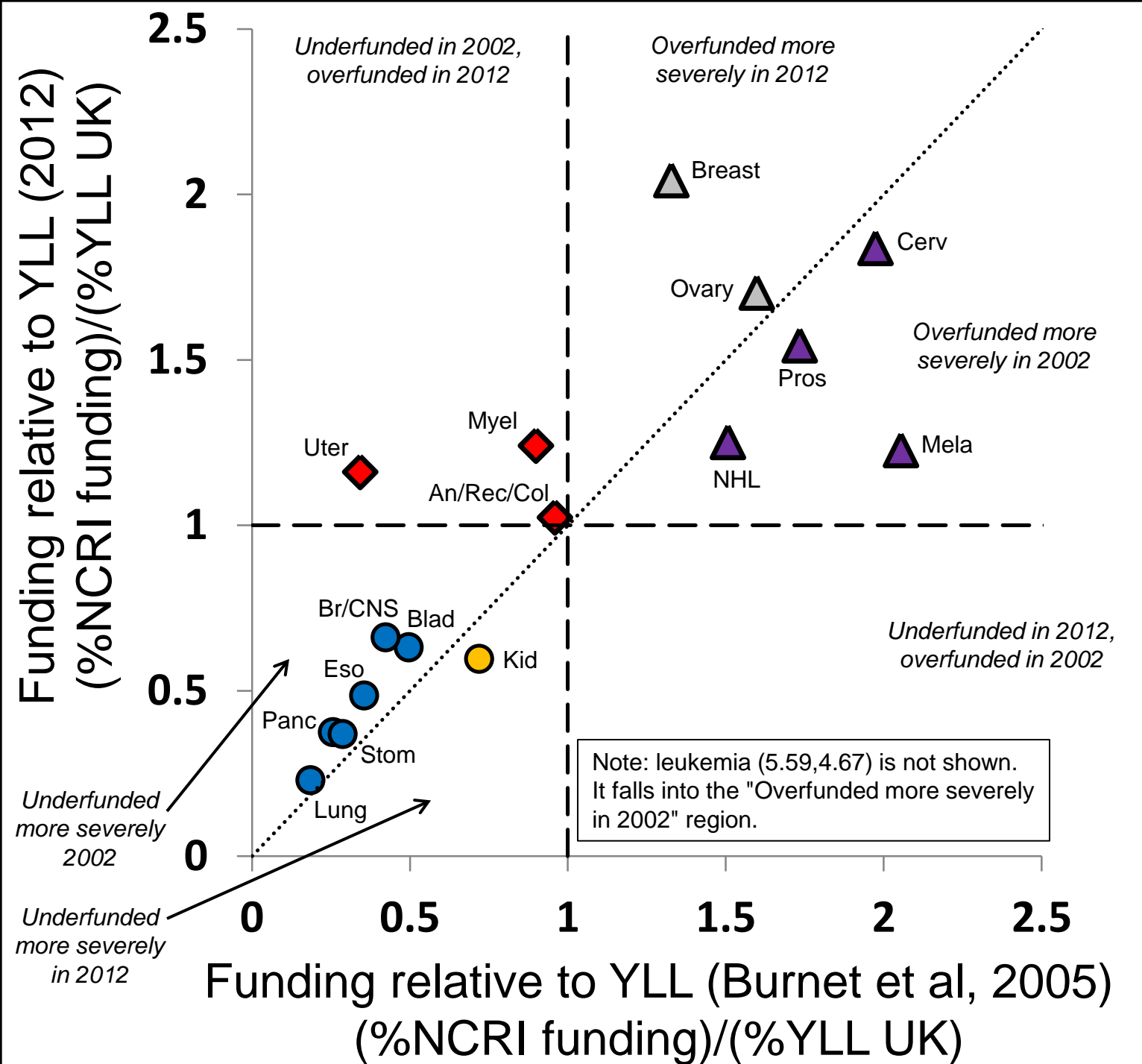

Supplement: Additional file 1: — Analysis of the research funding to years of life lost ratios for United Kingdom data presented in Table 2 on the Y-axis compared to 2002 data [ 3 ] on the X-axis, including lung cancer values. (PDF 89 kb) [file 12961_2015_50_MOESM1_ESM.pdf]

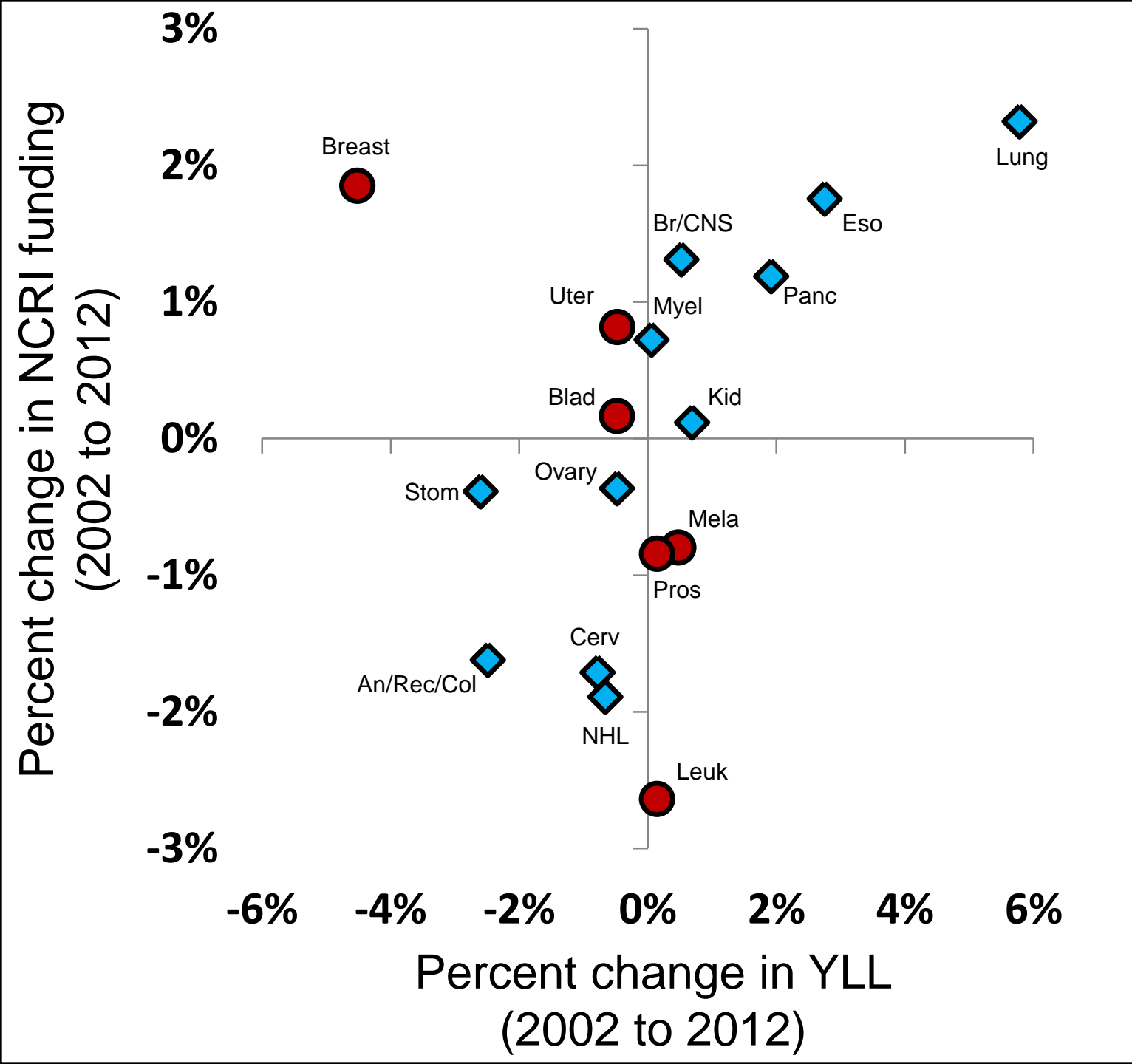

Supplement: Additional file 2: — Analysis of a comparison of the percentage differences in research funding (Y-axis) and years of life lost (X-axis) for the United Kingdom between this study and the values reported for 2002 [ 3 ], including lung cancer values. (PDF 100 kb) [file 12961_2015_50_MOESM2_ESM.pdf]

%YLL UK (this study)

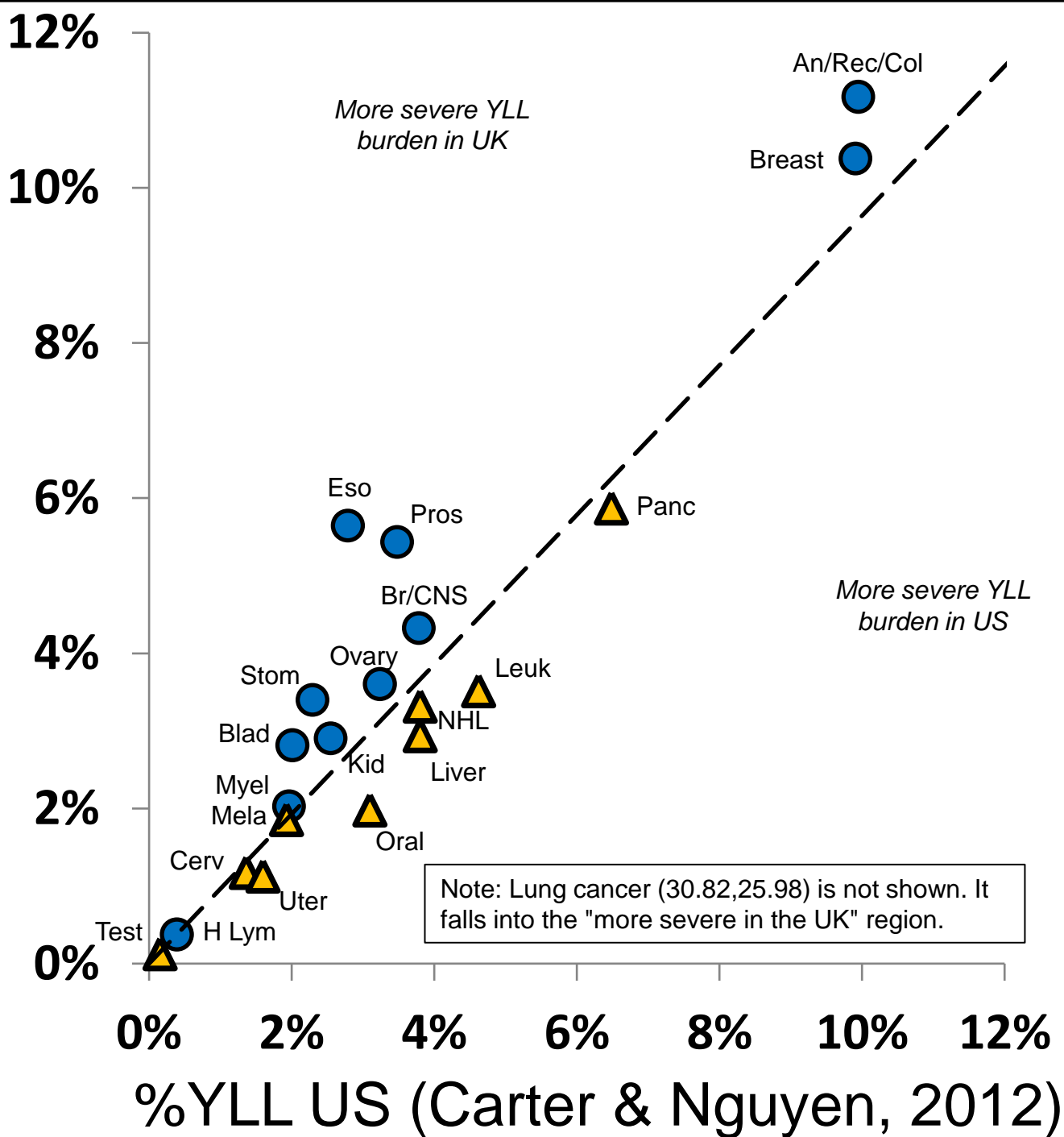

Supplement: Additional file 3: — Analysis of the years of life lost burden values for the United Kingdom on the Y-axis compared to a recent report of the same cancer types for the United States [ 10 ], including lung cancer values. (PDF 103 kb) [file 12961_2015_50_MOESM3_ESM.pdf]

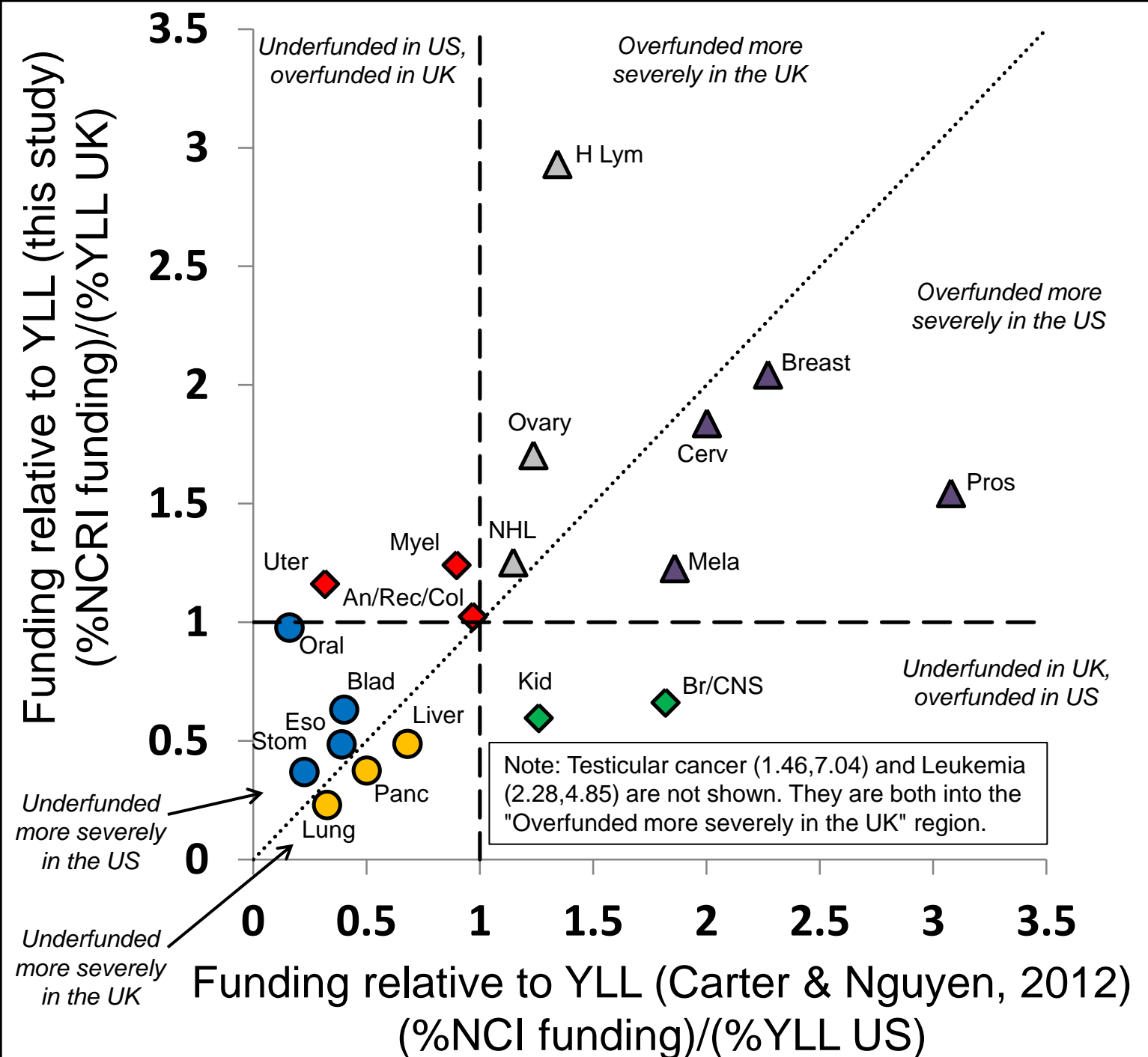

Supplement: Additional file 4: — The research funding to years of life lost ratios for United Kingdom data presented in Table 2 on the Y-axis compared to a recent report of the same cancer types for the United States [ 10 ] on the X-axis, including lung cancer values. (PDF 103 kb) [file 12961_2015_50_MOESM4_ESM.pdf]
